# An inclined tower crane suited to bridge tower construction

**DOI:** 10.1038/s41598-022-26222-x

**Published:** 2022-12-19

**Authors:** Kun Zhang, Hui Wang, Yong Zhou, Kaiqiang Wang, Xiaolin Fang, Baiben Chen, Dongdong Mu

**Affiliations:** China Construction Third Engineering Bureau Group Co., Ltd., Wuhan, 430000 China

**Keywords:** Mechanical engineering, Civil engineering

## Abstract

A new type of tower crane which could transform its tower body is presented. The inclined tower crane body can be tilted at some angle, which suits the construction of buildings with inclined architectural outlines, especially for the construction of bridge towers. The working principle and the design of the critical components are presented, two adjustable anchorages could pull the tower body section beneath the top anchorage closer to the construction region, and the related section of the tower body could change from vertical to inclined, while the free section of the tower body remains vertical. Finite element analysis is performed to compare with the traditional tower crane, the difference of the reaction force of the top anchorage is minor. Kinematic and dynamic analysis is carried out to simulate the working process of tilting, mechanical and kinematic parameters of key parts are obtained. At last, the experiment of a full-scale tower crane which could tilt 6° is carried out, and the experimental data validate the feasibility of this new type of tower crane.

## Introduction

As a main hoist transportation machinery, the tower crane plays an important role in the construction of bridges, high-rise buildings, cooling towers, etc. A tower crane always consists of a base, the tower body, anchorages, a slewing unit, a jacking unit, a driver cab, a jib, a counter jib, counter-weights, etc., the anchorages connect the tower body with the construction structure so that the height of the tower crane can be increased, and the tower body should keep vertical since installation.

Since the invention of the tower crane in the last century, the main structure of the tower crane has not been innovated, most of the recent research focus on the usage and layout on construction sites^1–4^, and the oscillation control of payload swing^5–10^, the main structural form of tower crane has not changed, the tower body is vertical, the anchorages are horizontal.

Due to structural considerations and aesthetic requirements, the profile of the construction structure is always inclined. During the construction process, the distance between the tower body and the construction region becomes bigger with the increase of construction height, and the bigger the distance is, the less weight the tower crane could hang up to the construction area, so a bigger tower crane is needed to lift the parts. What’s more, with the increasing height of construction, long anchorages are needed to support the tower crane, and the mounting of the long anchorage at a high altitude is quite burdensome and dangerous.

Especially during the construction of the bridge tower, to reduce the distance between the tower crane and the bridge tower construction area, one approach is to locate the tower crane in the corner of the bridge tower, such as in the building of Yueqing Bay Bridge^11^, Qingshan Yangtze River Bridge^12^, Sutong Bridge^13^, Anqing Yangtze River Railway Bridge^14^, Huanggang Yangtze River Rail-cum-road Bridge^15^, et al. This method could reduce the distance between the tower crane and the construction tower region to a certain extent, but it causes difficulties to the manufacturing and amounting of anchorages, and the mechanism of anchorages would be quite complicated.

Another approach is to locate a tower crane in the middle of the two bridge towers along the bridge’s longitudinal direction, such as Chizhou Yangtze River Highway Bridge^16^ and Shele Bridge in Taiyuan^17^, this approach would affect the installation of steel box girders, and the tower needs to be removed before the installation of steel box girders.

The third approach is to reinstall another tower crane before the mounting of the steel anchor box, Yangtze River highway bridge of Hubei East demolished one previous 1 600 KN.m tower crane before mounting the steel anchor box, then install one 11 000 KN.m tower crane to hang up the 29.14 t steel anchor box^18^. Shanghai-Suzhou-Nantong Yangtze River Bridge installed a 27 000 KN.m tower crane in the middle section of the tower body^19,20^, and the tower crane base anchoring area of the bridge tower body needs to be reinforced. This method would affect the construction schedule and increase the project price.

**Figure 1:**
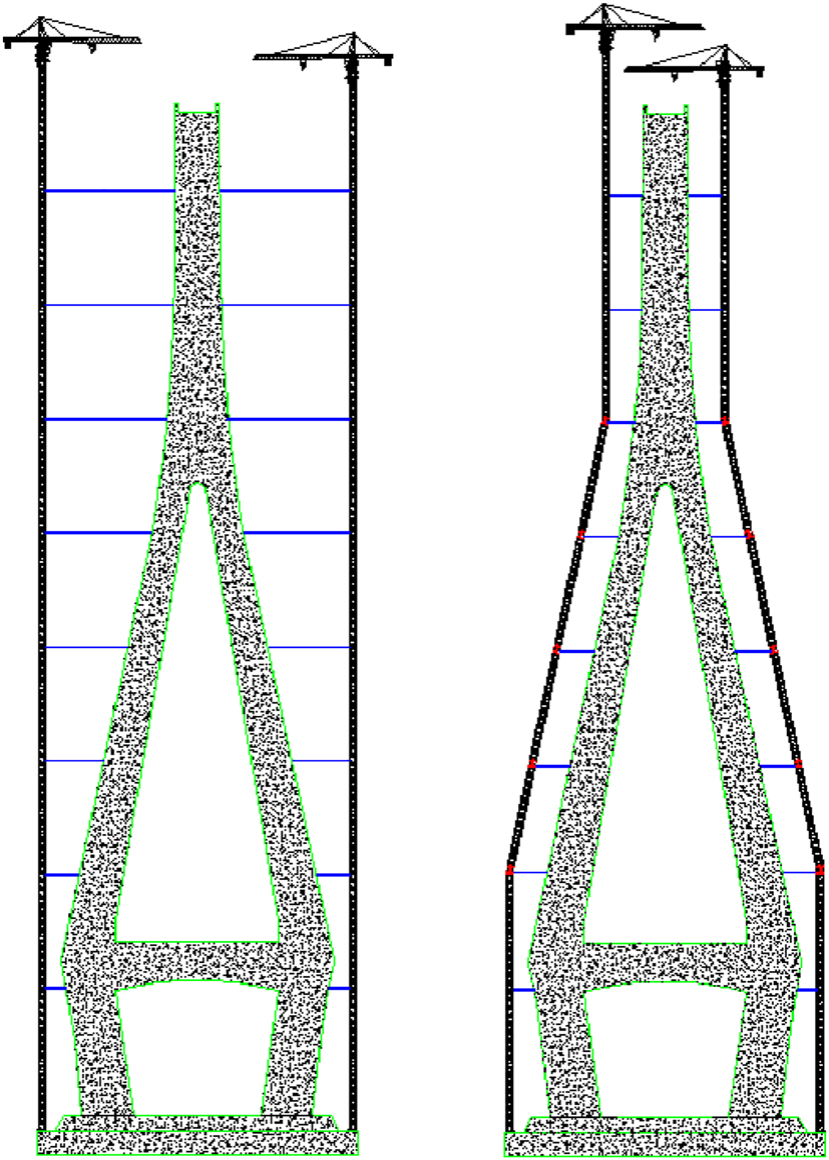
The inclined tower crane and the traditional tower crane (AutoCAD 2020).

This paper presents a type of tower crane which could change the traditional vertical tower body shape to fit the inclined architecture outline, as shown in Fig. 1. The new approach could keep the tower crane close to the construction area and shorten the anchorage length, which could make the tower crane more effective to hang up heavier weights to the top construction region, and a smaller tower crane can satisfy the lifting of the steel box girders. In addition, this method can reduce the risk in the high-altitude mounting of long tower crane anchorages.

## Machine design

### Prototype layout and working principle

**Figure 2:**
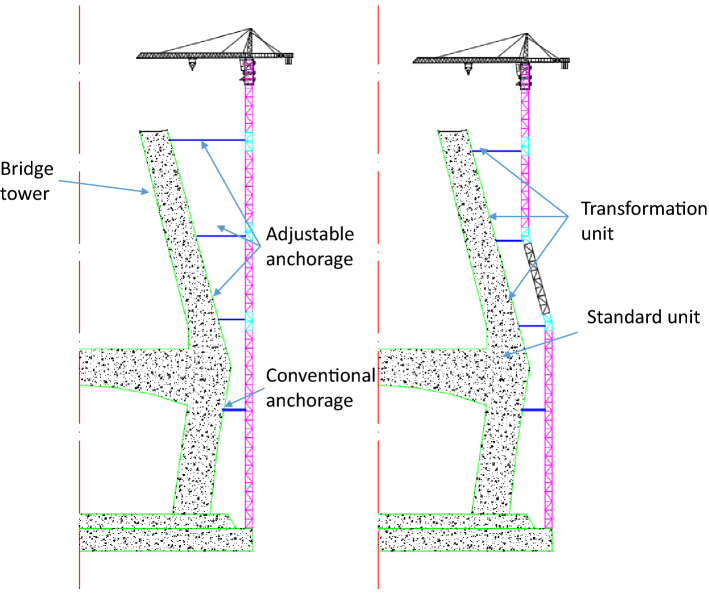
Composition with the inclined tower crane (AutoCAD 2020).

Compared to the traditional tower crane, the upper parts of the inclined tower crane include a crane jib, a counter jib, a slewing ring, a lifting sleeve frame, etc. These parts are the same, and the only differences are the tower body and the anchorages. As shown in Fig. 2, the inclined tower crane is comprised of standard units, transformation units, conventional anchorages, adjustable anchorages and the top section structure.

**Figure 3:**
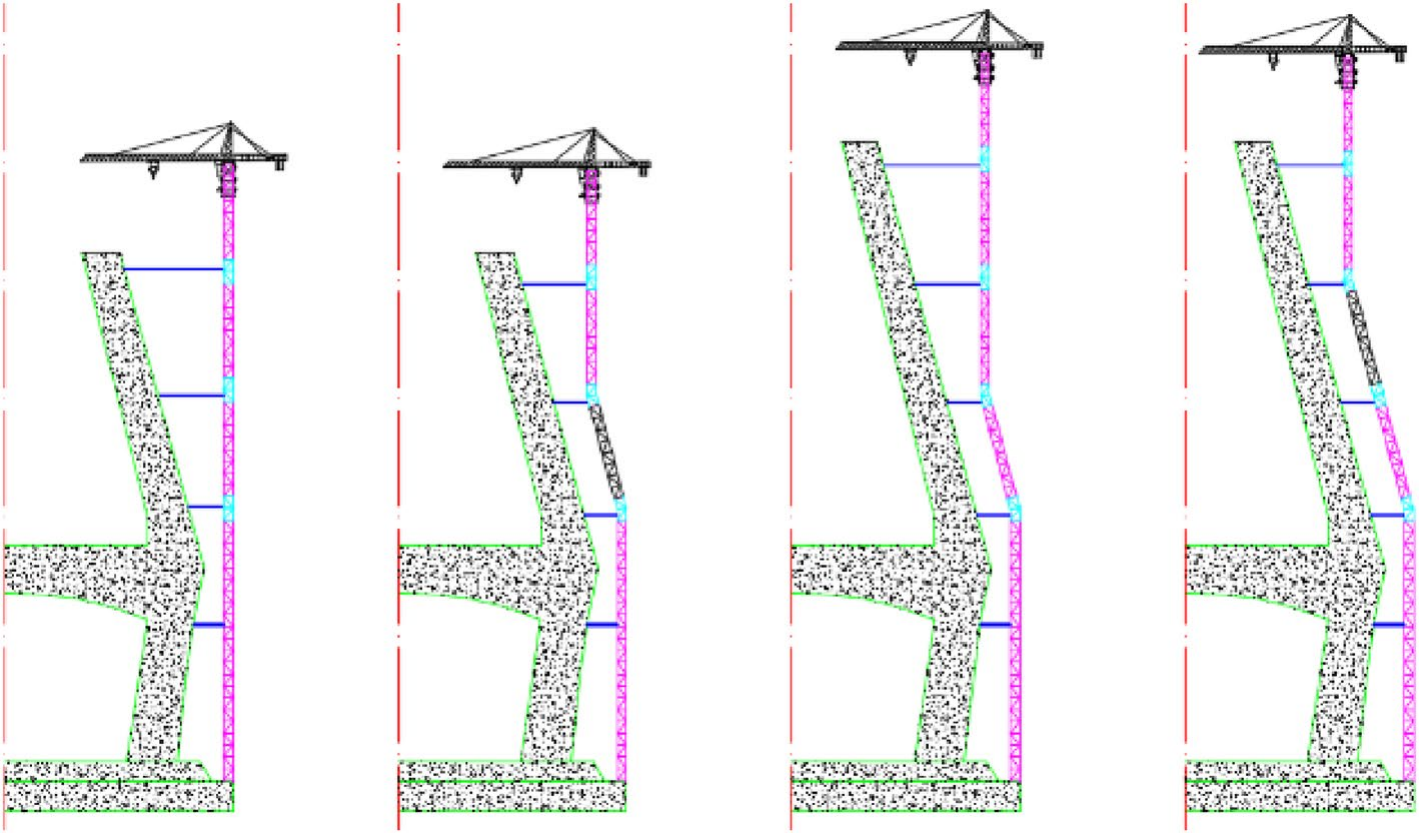
Working principle of the inclined tower crane (AutoCAD 2020).

The top two adjustable anchorages could drive the related transformation units to rotate, as shown in Fig. 3, and the two related transformation units rotate reversely, then the section of the tower body beneath the second (from top) adjustable anchorage could transform to an inclined shape, while the free section of tower body always keeps vertical during this period.

### Transformation unit design

The outline size of the transformation unit is the same as the standard unit, it’s a part of the tower body. What’s different is that the transformation unit can rotate by a certain angle.

**Figure 4:**
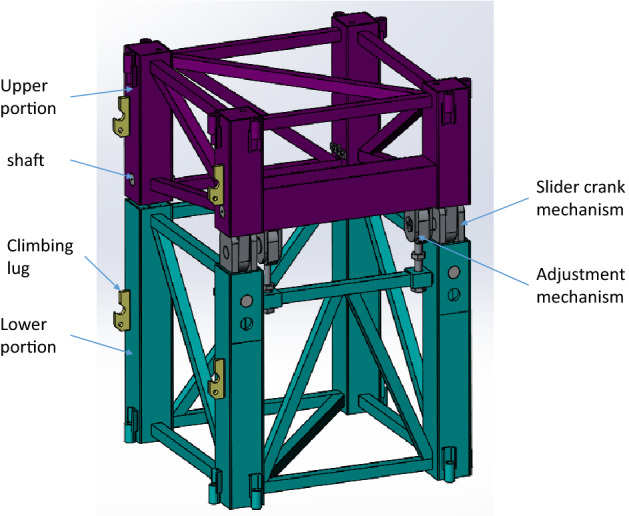
Structure of the transformation unit (Creo Parametric 9.0.1.0 Trial).

As presented in Fig. 4, the transformation unit is mainly composed of an upper portion, a lower portion, shafts, two slider crack mechanisms and two adjustment mechanisms, one end of the upper portion and one end of the lower portion are connected with the shaft, and the other ends of the two parts are connected by a slider-crank mechanism and an adjustment mechanism. The upper portion and the lower portion of the transformation unit mainly consist of vertical beams, horizontal rods and oblique rods, etc.

**Figure 5:**
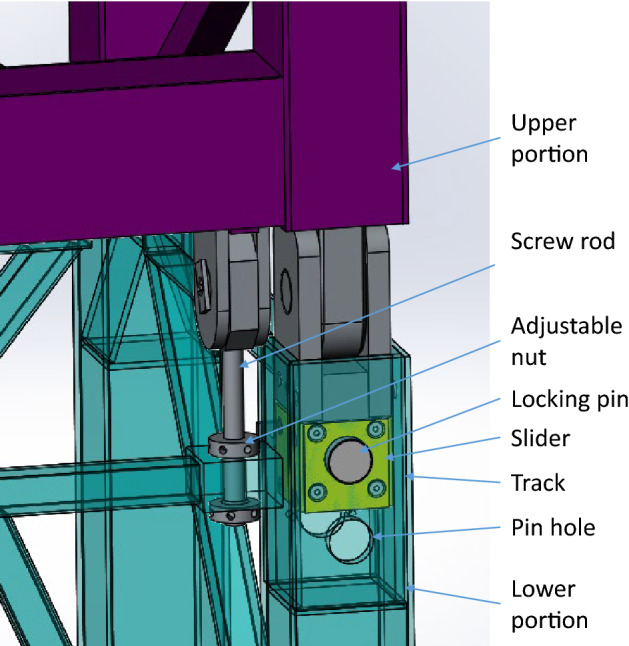
Slider crank mechanism and adjustable mechanism (Creo Parametric 9.0.1.0 Trial).

When the upper portion rotates around the shaft, as presented in Fig. 5, the slider could move in the track of the lower portion until the pin holes of the slider align with the holes of the lower portion, and the locking pins can be inserted, then the transformation unit can’t rotate. Copper blocks are mounted on the outside of the slider to reduce the friction of the motion.

The adjustment mechanism utilizes the screw-nut principle to adjust the position of the upper portion of the transformation unit, the pin holes can be aligned precisely, and pins can be thrust into the pin holes, then the transformation unit can’t rotate.

There are two adjustable nuts for an adjustable mechanism, and the adjustable nuts can move up and down on the screw rod. The adjustable mechanism has three main functions: First, the upper and lower nuts are positioned to limit the angle of the rotation, to ensure that the rotation will not exceed the set range; Second, when installing the locking pin, the position of the pin and the pin hole can be fine-tuned by the rotation of the nut, so that the locking pin and the pin hole are aligned precisely to facilitate the installation and removal of the locking pins; The third function is to ensure the structural safety of transformation unit, even when the locking pins are removed.

**Figure 6:**
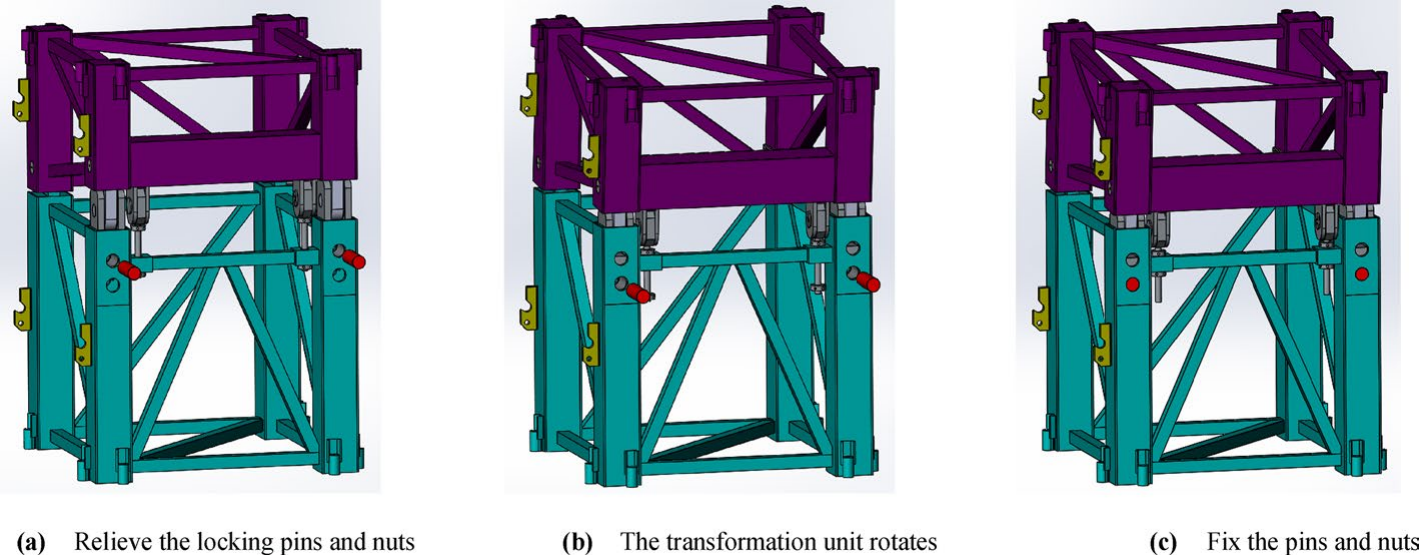
The workflow of the transformation unit (Creo Parametric 9.0.1.0 Trial). (**a**)Relieve the locking pins and nuts. (**b**) The transformation unit rotates. (**c**) Fix the pins and nuts.

The operation procedure of the transformation unit to change the angle is as follows, as presented in Fig. 6:

- Before the tilting of the inclined tower crane, remove the locking pins and move the upper nut to the top limit position, then the transformation unit could rotate;
- With the tilting motion of the transformation unit, the pin holes of the upper portion of the transformation unit would be aligned with the pin holes of the lower portion;
- Move the lower nut up to tighten the upper portion of the transformation unit, and utilize the lower nut and upper nut to align precisely the pin holes of the upper portion and the lower portion, and at last, insert the locking pins into the pin holes.

### Adjustable anchorage design

**Figure 7:**
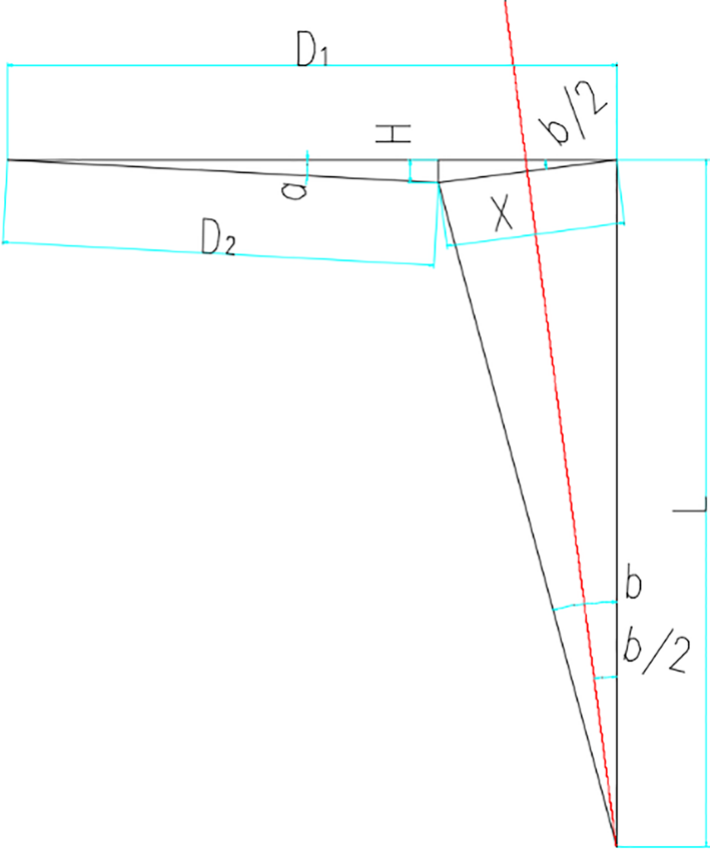
Simplified working model of the inclined tower crane (AutoCAD 2020).

As the deformation of the tower crane body is driven by the top two adjustable anchorages, the working principle can be simplified as shown in Fig. 7. The length of the deformable tower body is denoted as *L*, the length of anchorage after deformation is denoted as *D*, *D*_*1*_ is the length of the anchorage before tilting, *D*_*2*_ is the length of the anchorage after tilting, and b indicates the deformable tower crane body angle, while a is the rotated angle of anchorage after deformation, *X* and *H* represent the displacement and height change of the junction of the tower crane body and the anchorage meanwhile.

Through the above Fig. 7, the following relations can be got:

1 $$X=2Lsin(\frac{b}{2})$$

2 $$\begin{aligned} H & = 2L\sin \left( \frac{b}{2} \right)^{2} \\ & = 2L\left( {\frac{1 - \cos b}{2}} \right) = L\left( {1 - \cos b} \right) \\ \end{aligned}$$

3 $$\begin{aligned} D_{2} & = \left[ {H^{2} + \left( {D_{1} - X\cos \frac{b}{2}} \right)^{2} } \right]^{0.5} \\ & = \left[ {L^{2} \left( {1 - \cos b} \right)^{2} + \left( {D_{1} - L\sin b} \right)^{2} } \right]^{0.5} \\ \end{aligned}$$

4 $$\frac{X}{sin\left( a \right)} = \frac{{D_{2} }}{{sin\left( \frac{b}{2} \right)}}$$

**Figure 8:**
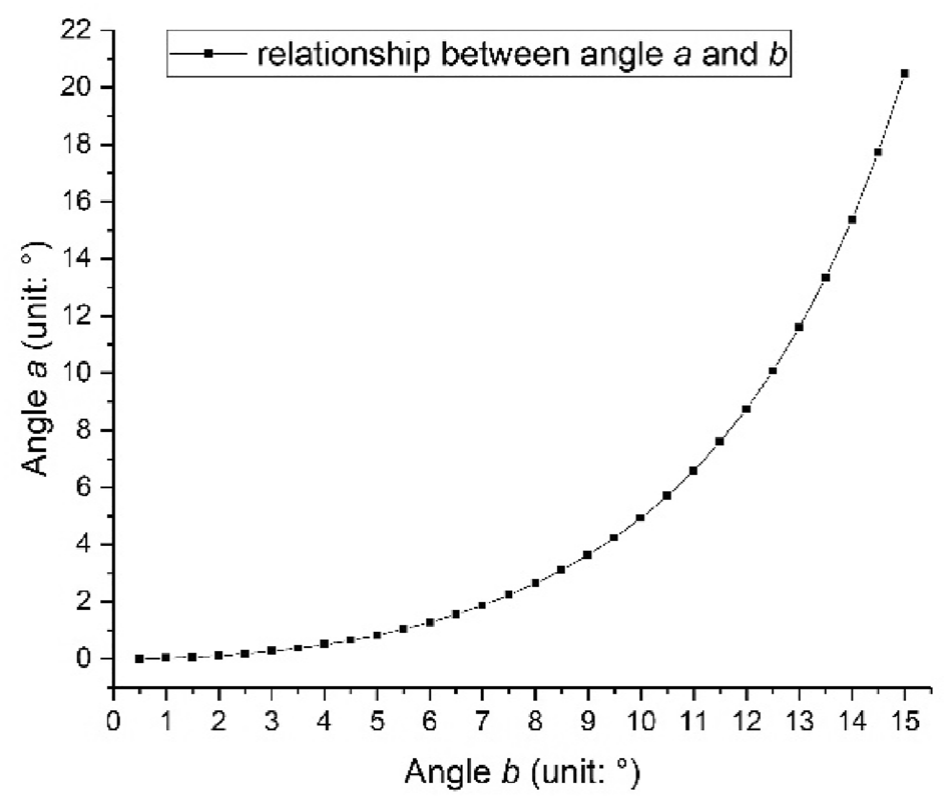
Relation of the adjustable anchorage rotating angle and the tower body transformation angle.

Assuming the initial anchorage length is 7m, and the length of the deformable tower crane body is 20m, the relationship between the rotating angle of the anchorage and the deformation angle of the tower crane body can be got through calculation, as shown in Fig. 8, and the rotating angle of the anchorage is affected by initial anchorage length, length of the deformable tower crane body and deformation angle of the tower crane body.

**Figure 9:**
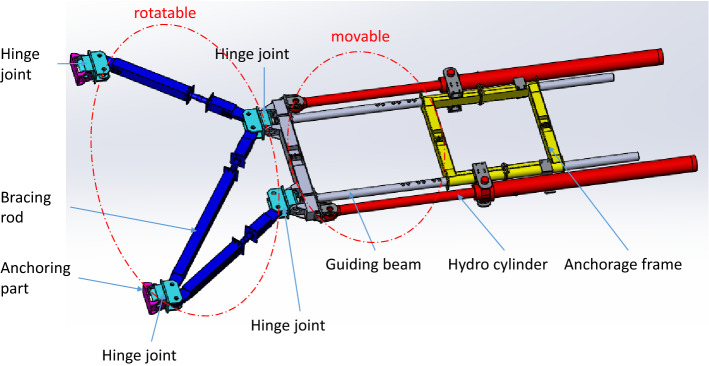
Structure of the adjustable anchorage (Creo Parametric 9.0.1.0 Trial).

Based on the analysis, not only could the anchorage shrink, but also could rotate at a certain angle. The structure of adjustable anchorage is shown in Fig. 9, the anchorage frame could move along two guiding beams driven by two hydro cylinders, and the two ends of three bracing rods could rotate vertically.

The two hydro cylinders and hydraulic control system could be reused for the next adjustable anchorage, so during the whole usage period of the inclined tower crane, only 4 hydro cylinders are needed. For every adjustable anchorage, the two hydro cylinders are driven by a hydraulic power pack, the hydraulic power pack could also be reused for the next adjustable anchorage.

**Figure 10:**
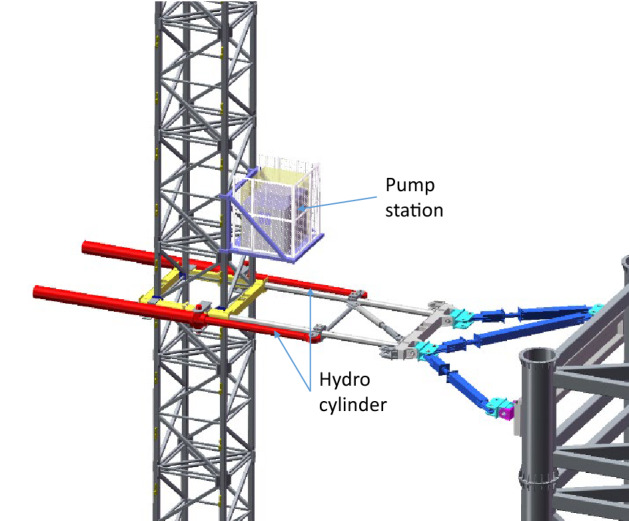
Location of the pump station (Creo Parametric 9.0.1.0 Trial).

In order to shorten the length of the hydraulic pipe between the pump station and the cylinder, as well as to facilitate the maintenance of the hydraulic system, the pump station is installed on the standard unit of the tower near the hydraulic cylinder, as shown in Fig. 10. The pump station is mounted on a station frame which is fixed to the standard unit of the tower crane with screws.

## Static analysis

To compare the difference between the inclined tower crane and the traditional tower crane, both two models are established and analyzed using finite element analysis.

Tower crane TC6013-8 (Joinhand Construction Machinery Co., Ltd.) is chosen for design and experiment. The inclined tower crane comprises several standard units, two transformation units and two adjustable anchorages. The size of the standard unit is 1.8 x 1.8 x 2.8 m, the heights of the two crane bodies are both 70 m, the height of the top anchorage is 20 m, and the height of the bottom anchorage is 40 m, the length of the top adjustable anchorage after shrinkage is 4m, and the inclined angle of the tower body is 6°.

### Mathematical model

**Figure 11:**
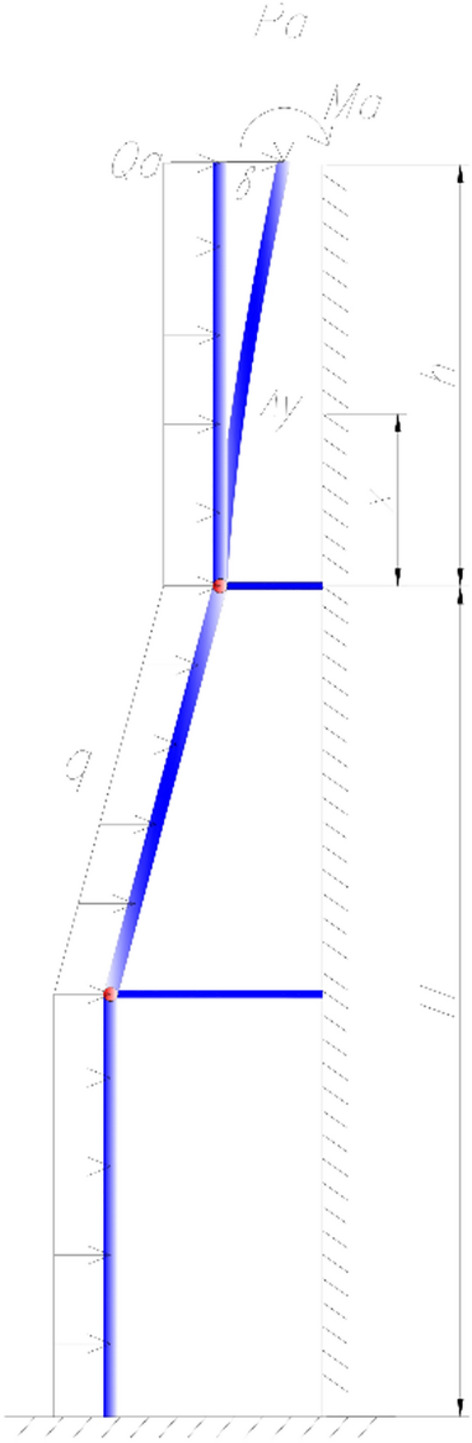
Deformation and deflection analysis diagram (AutoCAD 2020).

The inclined tower crane needs to have sufficient strength, stability and stiffness during daily use. In order to further analyze the force and deformation trend of the structure of the inclined tower crane under the action of lifting weight load and wind load, the deformation and deflection analysis diagram of the tower crane under the working condition is established^21–23^, as shown in Fig. 11. The vertical load of the tower crane is denoted as ${P}_{a}$, the bending moment at the top of the tower crane is denoted as ${M}_{a}$, ${Q}_{a}$ is the horizontal load at the top of the tower crane, *q* is the horizontal force of the wind load, $x$ and $y$ are Horizontal and vertical displacements.

Critical parameters include the height of the free section of the tower crane (*h*) and the height of the tower body (*H*) beneath the top anchorage.

The differential equation for the tower crane deflection is as follows:

5 $$EI\ddot{\Delta y}={M}_{a}+{P}_{a}(\delta -\Delta y)+{Q}_{a}(h-x)+\frac{q{(h-x)}^{2}}{2}$$

6 $${k}^{2}=\frac{{P}_{a}}{EI}$$

Then Eq. (5) can be expressed as

7 $$\ddot{\Delta y}+{k}^{2}\Delta y=\frac{{k}^{2}}{{P}_{a}}\left[{M}_{a}+{P}_{a}\delta +{Q}_{a}\left(h-x\right)+\frac{q{\left(h-x\right)}^{2}}{2}\right]$$

According to the calculus principle, the above equation is solved in general solution as

8 $$\begin{aligned} \Delta y & = Acoskx + Bsinkx + \frac{q}{{2P_{a} }}x^{2} - \frac{{Q_{a} + qh}}{{P_{a} }}x \\ & \quad + \frac{1}{{P_{a} }}\left( {M_{a} + P_{a} \delta + Q_{a} h + \frac{{qh^{2} }}{2}} \right) - \frac{q}{{P_{a} k^{2} }} \\ \end{aligned}$$

According to the boundary conditions, when $x=0$, $\Delta y=0$ and $\dot{\Delta y}=0$, It can be obtained from Eq. (8) that

9 $$A=\frac{q}{{P}_{a}{k}^{2}}-\frac{1}{{P}_{a}}\left({M}_{a}+{F}_{a}\delta +{Q}_{a}h+\frac{q{H}^{2}}{2}\right)$$

10 $$B=\frac{{Q}_{a}+qh}{{P}_{a}k}$$

According to the top boundary condition of the tower crane, when $x=h$, $\Delta y=\delta$, and assume $u=kh$, then the static horizontal displacement of the top of the tower crane can be obtained as

11 $$\begin{aligned} \Delta y_{1} & = \frac{{M_{a} }}{{P_{a} }}\left( {secu - 1} \right) + \frac{{Q_{a} }}{{P_{a} }}\left( {\frac{tanu}{k} - h} \right) \\ & \quad + \frac{q}{{P_{a} k^{2} }}\left( {1 - \frac{{u^{2} }}{2} + utanu - secu} \right) \\ \end{aligned}$$

### Load and condition

According to the specification of TC6013, the stress is bigger when the tower crane is out of service than in service, and this paper just considers the state of the tower crane out of service. The jacking unit, slewing rig, jib, counter jib driver cab and tower head are not modeled, just be considered as a vertical load of 400 kN in the Z direction (upright to the crane) based on the gravity, the bending moment is 2430 kN.m in the Y direction (lateral to the crane), and the horizontal force in the X direction (horizontal to the crane, mainly caused by wind) is 85 kN.

As the stress is affected by the direction of the crane jib, 0°, 45°, 90°, 135°, and 180° between the crane jib and the anchorage direction are taken into account in the analysis.

### Modelling of the tower crane

**Figure 12:**
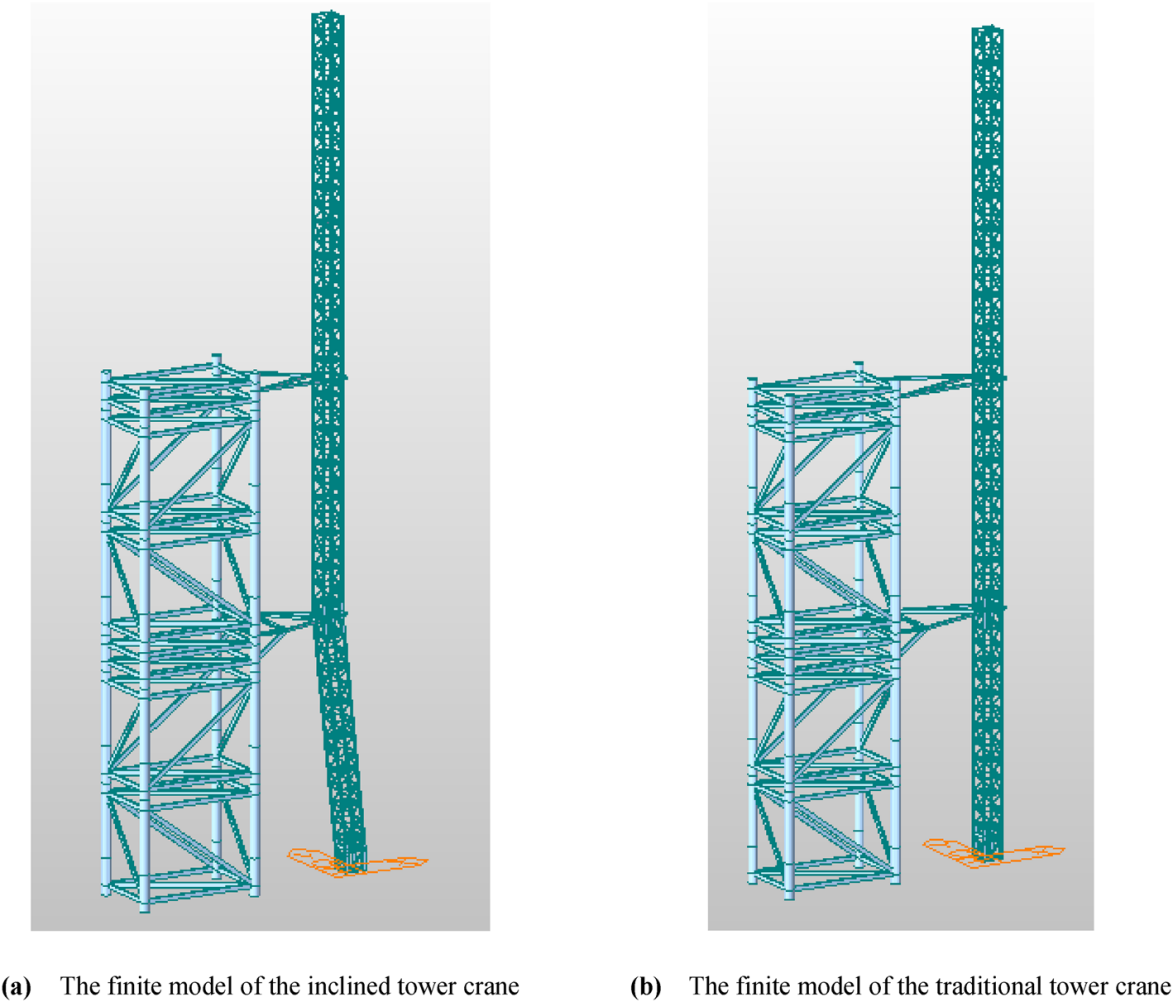
Finite element models of the inclined and the traditional tower crane (Midas Civil 8.3.2). (**a**) The finite model of the inclined tower crane. (**b**) The finite model of the traditional tower crane.

The inclined tower crane and the traditional vertical tower crane are modeled using 3D space truss elements, as shown in Fig. 12, the upper crane structure and weight, such as the climbing frame, the slewing ring, the slewing tower, the tower head, the operator cab, the jib, and the counter-weight, etc. are not modeled but considered as a vertical load.

To anchor the tower crane, a steel tower is designed and modeled, the height of the steel tower is 42.5 m, and the two adjustable anchorages are anchored to one side of the steel tower. The stiffness and robustness of the steel tower are big enough to support the tower crane.

### Stress and displacement analysis

**Figure 13:**
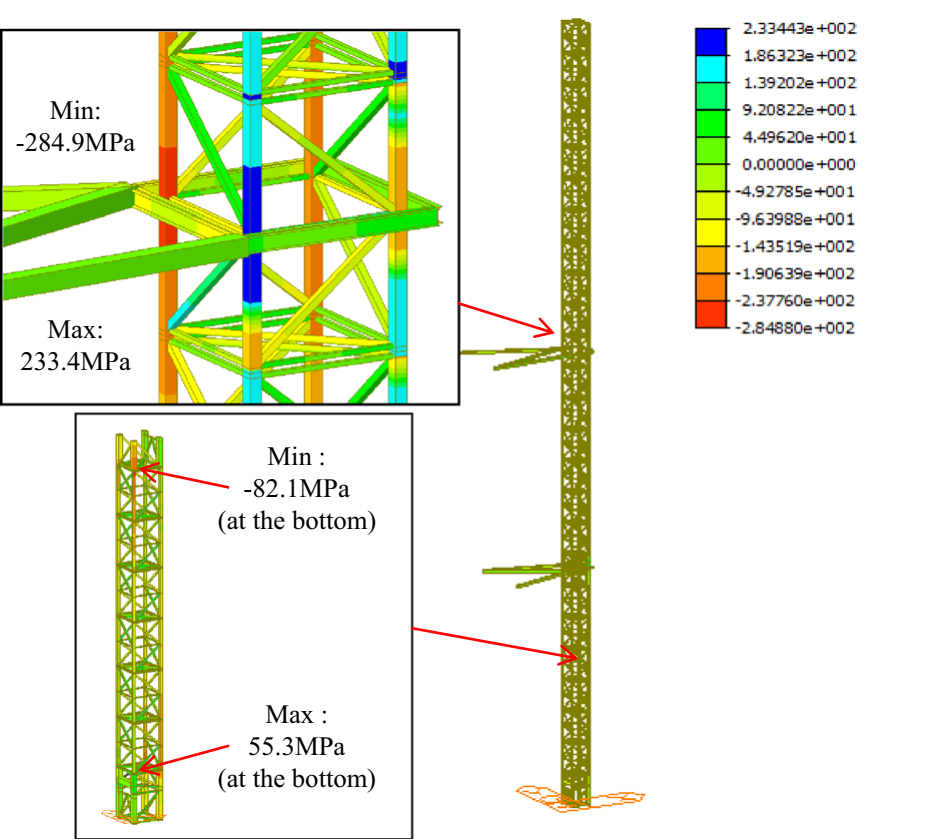
Stress analysis of the inclined tower crane (Midas Civil 8.3.2).

**Figure 14:**
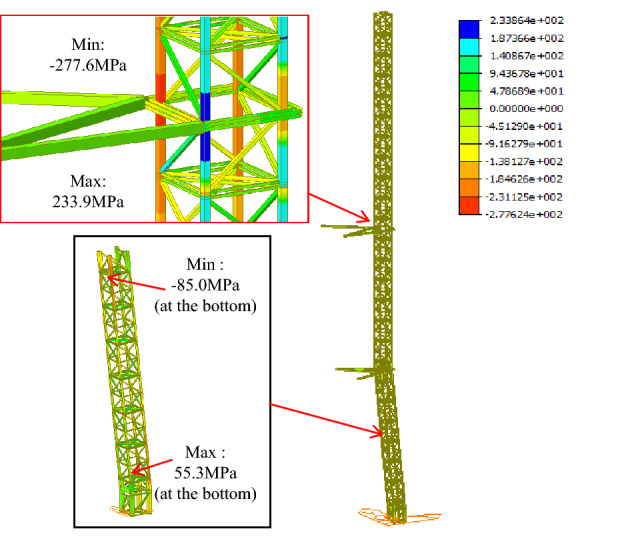
Stress analysis of the traditional tower crane (Midas Civil 8.3.2).

The model analysis is performed to compare the inclined tower crane and the traditional tower crane, as shown in Figs. 13 and 14, there is a litter difference both in stress and displacement, the maximum stress area is located at the bottom of the free section of the tower crane, the maximum stresses in the inclined and vertical states are 284.9 Mpa and 277.6 MPa respectively. Once the anchorages are assembled, the stress of the tower body beneath of anchorage is reduced to a lower level, the maximum stresses beneath the top anchorage in the inclined and vertical states are 82.1 Mpa and 85 MPa respectively.

To analyze the effect of the inclined tower body on the anchorages, the reaction forces of anchorages are extracted and compared between the inclined tower crane and the traditional tower crane, presented in Tables  1, 2, 3, 4 and 5, it can come to the following conclusions.

- Beneath the anchorage, the bending moment of the tower crane is mainly suffered by the top two anchorages, the stress of the tower body beneath the top anchorage is much smaller than the stress above the top anchorage.
- The reaction force of the top anchorage is bigger than other anchorages, and the stress of the lower anchorage decreases gradually.
- There is a quite small difference in the reaction force of the top anchorage between the inclined tower crane and the traditional tower crane, but a greater difference in the reaction force of the lower anchorage, for the reason of the inclined tower body.

**Table 1:** Reaction force of anchorage when JIB in 0°.

Item	Inclined tower crane. (in KN)			Traditional tower crane. (in KN)		
Direction	x	y	z	x	y	z
Top anchorage	242.0	0.7	− 33.2	239.6	0.9	-22.1
Bottom anchorage	− 21.2	0.1	− 5.7	− 50.4	− 0.8	− 9.4

**Table 2:** Reaction force of anchorage when JIB in 45°.

Item	Inclined tower crane. (in KN)			Traditional tower crane. (in KN)		
Direction	X	y	z	x	y	z
Top anchorage	177.6	163.3	− 26.8	175.7	160.7	− 28.7
Bottom anchorage	− 28.7	− 45.5	− 5.4	− 43.3	− 43.7	− 9.3

**Table 3:** Reaction force of anchorage when JIB in 90°.

Item	Inclined tower crane. (in KN)			Traditional tower crane. (in KN)		
Direction	x	y	z	x	y	z
Top anchorage	22.1	230.4	− 11.1	21.3	226.6	− 12.2
Bottom anchorage	− 54.6	− 63.5	− 4.3	− 42.9	− 60.6	− 9.3

**Table 4:** Reaction force of anchorage when JIB in 135°.

Item	Inclined tower crane. (in KN)			Traditional tower crane. (in KN)		
Direction	*x*	*y*	*z*	*x*	*y*	*z*
Top anchorage	− 133.5	162.8	4.5	− 133.0	160.0	− 5.0
Bottom anchorage	− 83.7	− 43.3	− 3.2	− 49.3	− 41.8	− 9.4

**Table 5:** Reaction force of anchorage when JIB in 180°.

Item	Inclined tower crane. (in KN)			Traditional tower crane. (in KN)		
Direction	x	y	z	x	y	z
Top anchorage	− 198.0	− 0.1	10.9	197.0	− 0.1	− 2.0
Bottom anchorage	− 99.0	3.4	− 2.6	− 58.8	1.9	− 9.5

## Kinematics and dynamic analysis

As the free section of the tower body needs to be vertical when the two adjustable anchorages drive the tower crane to be inclined. A kinematics model is established to simulate the working process, and the driving force of the two adjustable anchorages, the speeds of the two adjustable anchorages, the rotating angle of transformation units and the rotating angle of the two adjustable anchorages can be obtained from the simulation.

### Kinematics and dynamic model

**Figure 15:**
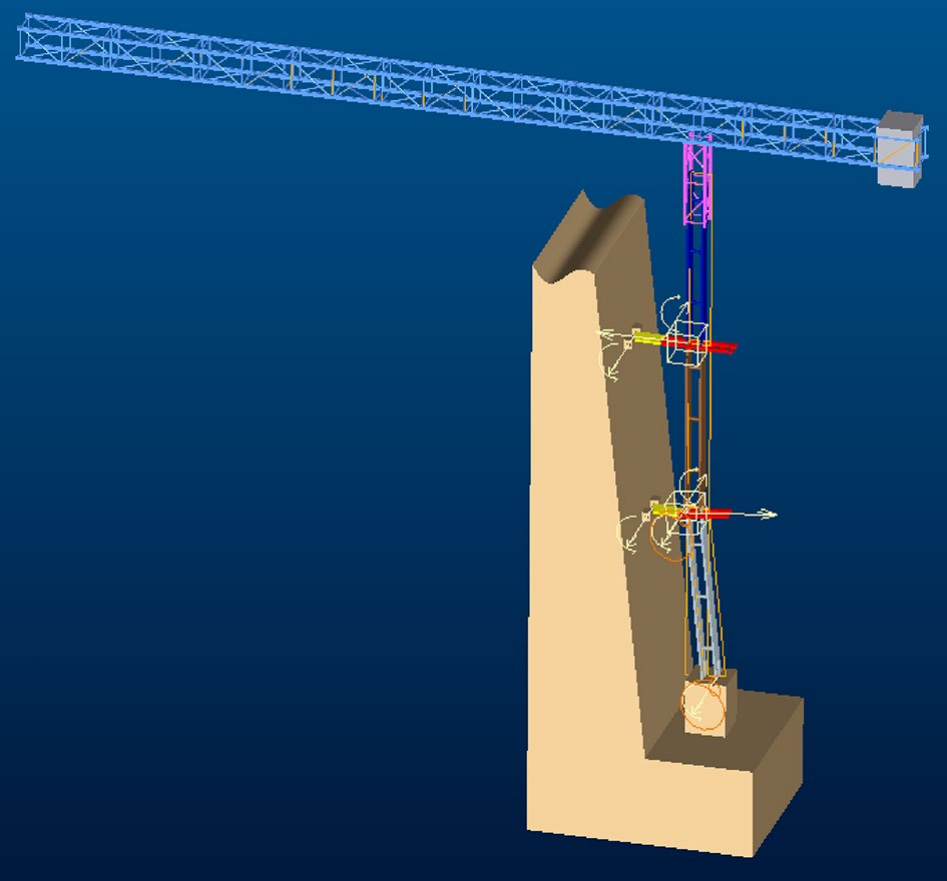
Kinematics Model (Creo Parametric 9.0.1.0 Trial).

The dynamic simulation of the whole mechanism is performed, as shown in Fig. 15, the two adjustable anchorages are anchored to the lateral bridge tower, and all the components apply gravity. The tower crane is in equilibrium before tilting, then the whole running process of the tower crane body transformation is simulated, and the kinematics and mechanical parameters of the key components can be monitored in the process.

### Key parameter analysis

Two servo motors are added to the tower crane body hinged axes, the directions of the two motors are reversed, and the amplitudes are the same, so the free section of the tower body would keep vertical, and then displacement sensors and force sensors are located on the two adjustable anchorages to measure the driving forces and shrinking speeds of the two anchorages.

**Figure 16:**
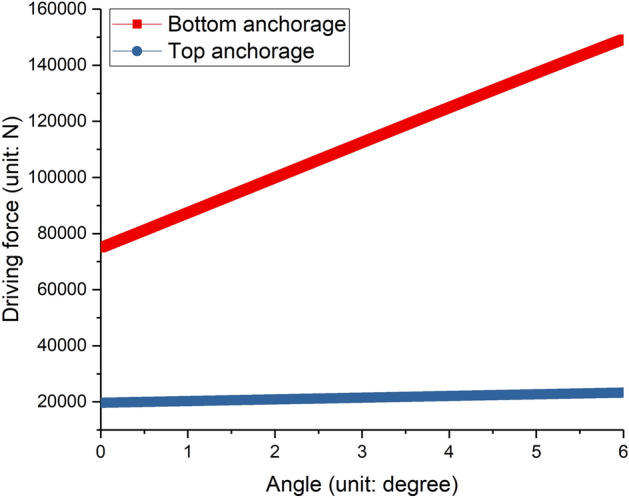
Driving forces of two adjustable anchorages.

The driving force of the two anchorages is shown in Fig. 16, and the driving forces both increase with the increase of the tilt angle, they are approximately linear. The driving force of the top anchorage is much smaller than that of the lower anchorage, in the initial state, the driving force of the top and bottom anchorage are 19.6 KN and 74.9 KN respectively, and with the increase of tilt angle, the driving force of the bottom anchorage rises rapidly, while the driving force of the top anchorage shows a slow increase trend. At the end of the movement, the driving force of the bottom anchorage reached 149.3 KN, an increase of 99.4%, while the driving force of the top anchorage is 23.2 KN, an increase of 18.4%. The reason for the rapid increase in the driving force of the bottom anchorage is that after the tower crane is tilted, the center of gravity of the top structure is shifted and a horizontal load will be generated on the bottom anchorage. In order to overcome the horizontal load, the driving force of the bottom anchorage increased significantly. However, the driving forces of the two attachments are significantly smaller than the reaction forces of the anchorages when the tower crane is in use.

**Figure 17:**
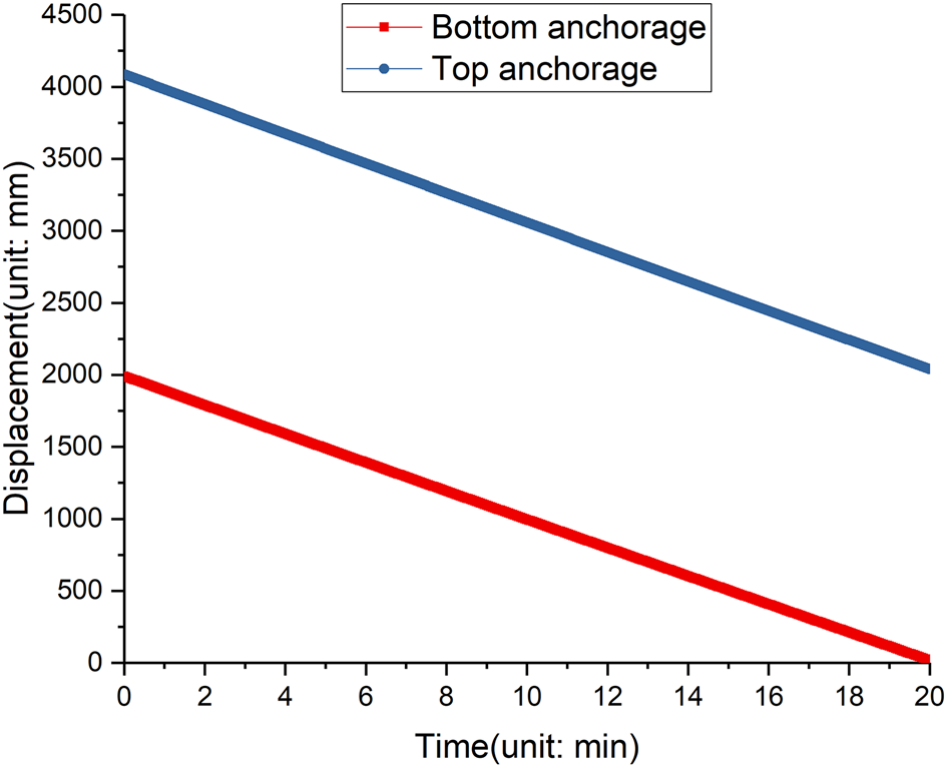
Shrinking speeds of two adjustable anchorages.

The speeds of the two anchorages shrinking are shown in Fig. 17, they are approximately linear, and have a small difference. The speed data can be exacted and input to the machinery control system, and control the hydro cylinder’s movement.

**Figure 18:**
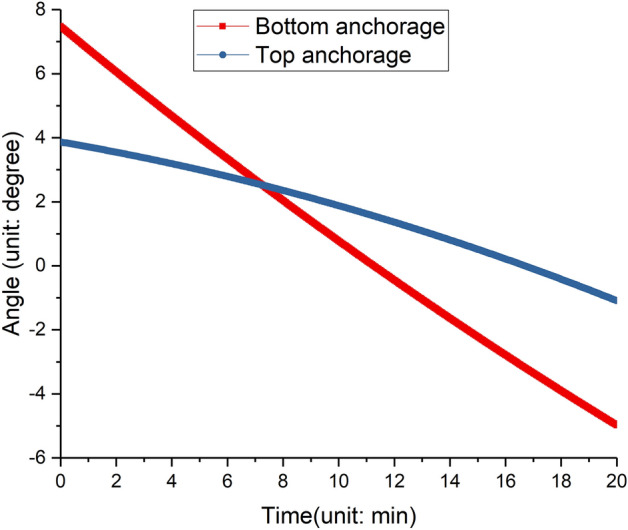
Rotating angles of two adjustable anchorages.

And Fig. 18 shows the rotating angles of anchorages, the rotating angle is related to the transformation angle, the distance between adjacent anchorages and the anchorage length. Initially, the adjustable anchorage is not horizontal, but with a giving upward angle to avoid the angle of the anchorage to be large after transformation. if the transformation angle *a*=6°, the length of the deformable tower body *L*=20 m, and the anchorage length after adjusting *D*_*2*_=4 m, then the rotating angle of the two anchorages are 6.46° and 4.95° respectively, the detailed angle information is shown in Table  6.

**Table 6:** Anchorage angle.

Item	Before transformation. (in °)	Before transformation. (in °)
Top anchorage	3.86	4.88
Bottom anchorage	− 1.09	− 1.58

## Experimental validation

To validate the design and analysis, a full-scale inclined tower crane was assembled and tested in Wuhan, China, mainly two types of experiments were conducted, one is the tilting test, and the other is the hoisting test. The monitoring system recorded the state data of key components, every hydro cylinder was equipped with one displacement sensor, strain sensors were placed on the key positions based on the calculation, angle sensors were placed on the transformation units and the adjustable anchorages, and one angle sensor was placed on the free section of the tower body to monitor the perpendicularity of the tower crane, what’s more, several acceleration sensors are placed on the tower body to monitored the shaking motion of the tower crane.

### Tilting test

Based on the simulation of the kinematic analysis of the inclined tower crane, the speed of four hydraulic cylinders could be obtained. By the control of the hydraulic system, the two adjustable anchorages shrink at different speeds simultaneously, the two transformation units rotate reversely, and the section of the tower body underneath the top anchorage could tilt slowly until the two transformation units rotate 6°.

**Figure 19:**
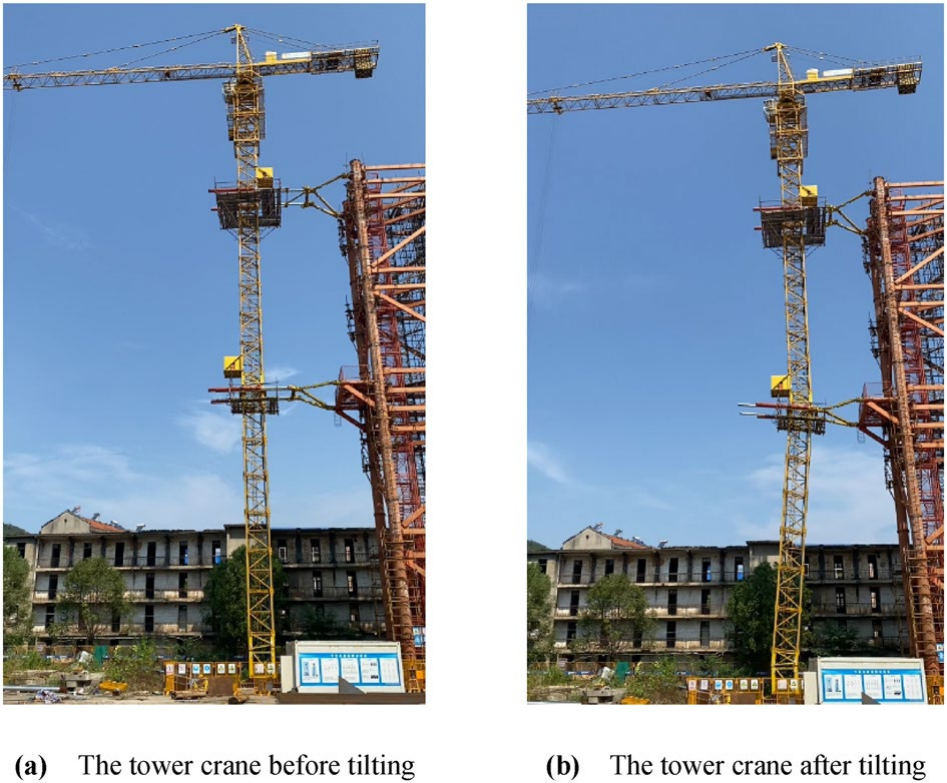
Tilting test of the inclined tower crane. (**a**) The tower crane before tilting. (**b**) The tower crane after tilting.

As shown in Fig. 19, the two adjustable anchorages drive the tower body from the vertical state to the inclined state, the tower crane is in balance before deformation, the wind scale doesn’t exceed Grade four, the whole working time is almost 20 minutes, then the two adjustable anchorages push the tower crane body from the inclined state to the vertical state, the perpendicularity of free section of tower body during the whole test is not bigger than 3.1/1000.

### Hoisting test

**Figure 20:**
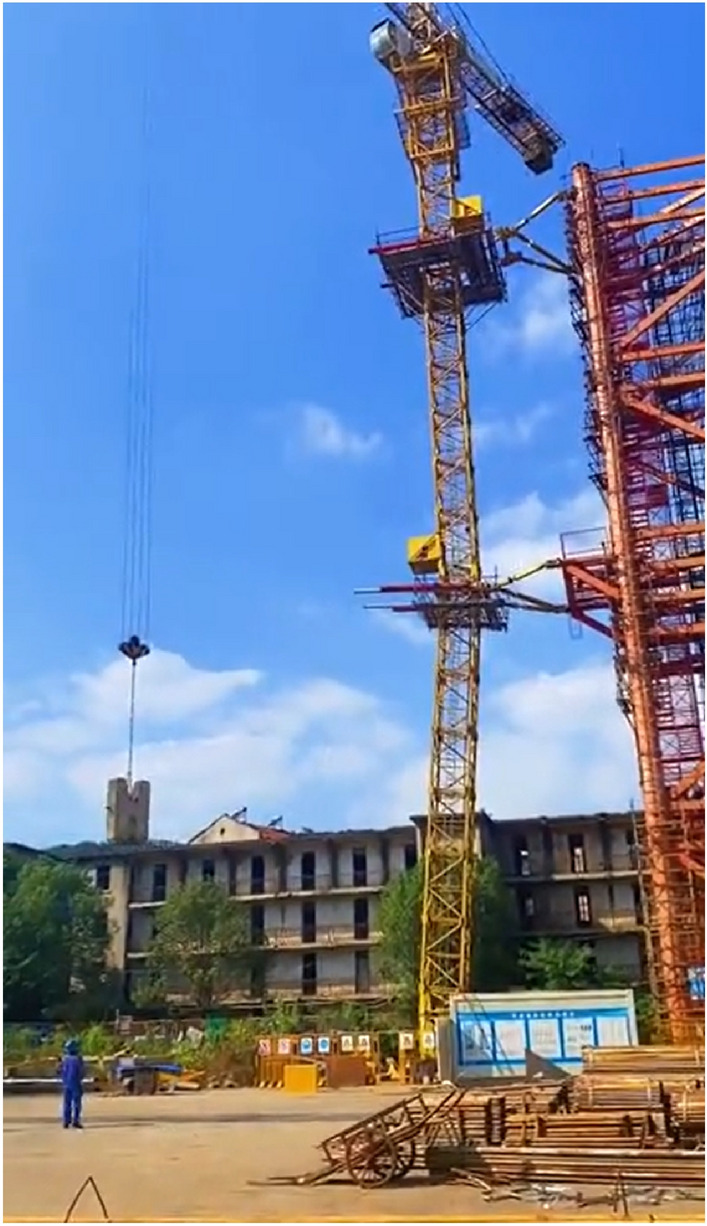
Hoisting test of the inclined tower crane.

In order to verify the lifting performance of the tower crane, the hoisting test was carried out, the manipulation of the inclined tower crane was controlled by the operator on the ground through a remote control handle, and there was no driver in the tower crane driver room. The maximum hoisting weight of TC6013 was 6 t, and the length of the jib of the experimental tower crane was 50 m, the hoisting weight was respectively 1 t, 2.3 t, 3.4 t and 6 t, as presented in Fig. 20, and movement of ascent, descent, rotation, and amplitude changing is tested during the experiment.

### Test results

According to the test process, the stress is monitored by applying strain gauges to key locations of the tower crane in the upright and tilted states under a 6 t load. The test results show that the maximum stress position is at the bottom of the free section of the tower crane, which is in accordance with the previous analysis. After analysis and comparison, the maximum calculated stress and test stress (4 points at the bottom of the free section of the tower body) comparison is shown in Table  7.

**Table 7:** Calculated stress and test stress.

Item	Simulation value in Upright state. (in MPa)	Test value in Upright state. (in MPa)	Comparison	Simulation value in inclined state. (in MPa)	Test value in inclined state (in MPa)	Comparison
Sensor 1	68.4	67.2	− 1.80%	68.6	64.5	− 6.00%
Sensor 2	− 73.1	− 78.8	7.80%	− 72.6	− 75.7	4.30%
Sensor 3	62.6	68.5	9.40%	58	65.8	13.40%
Sensor 4	− 58.6	− 60.5	3.20%	− 58.6	− 58.1	− 0.90%

From the above table, it can be obtained that there is almost no difference between the maximum stress value of the section in the upright state of the tower crane and the tilted state of the tower crane, so it shows that the tilted tower has little effect on the stress of the tower crane. And comparing the calculated value and the test value shows that the maximum difference percentage is 13.4%, and the simulation results are credible.

## Conclusions

In this study, we present a novel tower crane that could change the tower crane body shape to inclined, the freedom section of the tower body remains vertical, during the construction of the architecture with the inclined outline, this approach could reduce the distance between the tower crane body and the building construction region, shorten the length of the anchorages, improve the working efficiency of tower crane and reduce the difficulty and risk of the long anchorage installation at height.

The key components of the inclined tower crane include the transformation units and the adjustable anchorages, the transformation unit can rotate and the adjustable anchorage can stretch out and draw back, the transformation units are located on the position of the tower body which is connected with the adjustable anchorages. With the shrinkage of the adjustable anchorage, the related transformation would rotate by a certain angle.

The static simulation shows the stress of the tower body beneath the top anchorage is much smaller than the freedom section of the tower body, for the reaction force of the anchorage, the lower anchorages are much smaller than the top anchorage, and there is a small difference for the top anchorage reaction force between inclined tower crane and traditional tower crane.

The kinematic analysis of the entire inclined tower crane model was carried out to simulate the whole working process of tilting, key parameters such as the driving force of the two adjustable anchorages, the rotating speed of transformation units, the shrinking speed of adjustable anchorages and the rotate angle of adjustable anchorages can be obtained.

At last, the inclined tower crane verification experiments, including tilting and hoisting, were conducted in Wuhan, China, and experiment results proved the feasibility and reliability of the design.

## Data Availability

The datasets used and/or analysed during the current study are available from the corresponding author on reasonable request.
